# Using a Blood Biomarker to Distinguish Benign From Malignant Pulmonary Nodules: A Subgroup Analysis Comparing Screen Detection, Sex, Smoking History, and Nodule Size

**DOI:** 10.1016/j.chest.2023.06.037

**Published:** 2023-07-05

**Authors:** Kathryn J. Long, Trevor Pitcher, Jonathan S. Kurman, Michael A. Pritchett, Gerard A. Silvestri

**Affiliations:** a Medical University of South Carolina, Charleston, SC; b Biodesix, Inc., Boulder, CO; c Medical College of Wisconsin, Milwaukee, WI; d FirstHealth of the Carolinas & Pinehurst Medical Clinic, Pinehurst, NC

To the Editor:

An estimated 1.5 million pulmonary nodules are identified in the United States annually, with a prevalence of malignancy of 5%, which rises to 25% by the time patients are referred to a pulmonologist.^1^^,^^2^ Current guidelines recommend evaluating the pretest probability of cancer for indeterminant pulmonary nodules using clinical experience and validated clinical risk prediction models to determine next steps in management.^3^ Low-risk nodules (probability of cancer, < 5%) are managed with surveillance, whereas those at higher risk undergo functional imaging, biopsy, or surgery. Unfortunately, in current practice many patients undergo invasive testing for benign disease, including 22% of screen-detected nodules.^2^^,^^4^^,^^5^ A rule-out biomarker could aid in risk stratification by determining which nodules can be managed with surveillance, thereby preventing unnecessary invasive procedures.

A proteomic integrated classifier (IC) that combines two plasma proteins (LG3BP and C163A) with five clinical and imaging factors (age, smoking status, nodule size, edge, and location) previously was shown to assist in identifying benign nodules with a negative predictive value (NPV) of 98%.^6^ It is estimated that use of the biomarker in clinical practice could decrease the rate of invasive testing by as much as 40%.^6^ We undertook this study to evaluate the performance of the IC among various subgroups of patients using two large prospective cohorts based on screen detection, sex, smoking status, and nodule size.

## Methods

The Pulmonary Nodule Plasma Proteomic Classifier (PANOPTIC) trial (ClinicalTrials.gov Identifier: NCT01752114) was a prospective, multicenter, observational study to evaluate the clinical performance of the IC Nodify XL2 test (Biodesix, Inc.). Full details of the trial have been published previously.^6^ Briefly, included patients were aged 40 years or older with a newly detected nodule 8 to 30 mm in diameter. Benign diagnosis was determined by histopathologic analysis, radiographic resolution, or stability over 1 year and malignant diagnosis was determined by histopathologic analysis. Patients with a Mayo solitary pulmonary nodule probability of cancer of ≤ 50% were included. The Observational Registry Study to Evaluate the Performance of BDX-XL2 in Routine Clinical Use (ORACLE) trial (ClinicalTrials.gov Identifier: NCT03766958) was a multicenter, prospective, observational study to evaluate the clinical usefulness of the IC. Inclusion criteria were similar in both trials. For the present study, the two cohorts were pooled together and the performance of the IC was assessed using sensitivity, specificity, and NPV based on subgroups including sex, smoking status, and nodule size. To compare screen vs incidentally detected nodules, only the ORACLE cohort was included because PANOPTIC was performed before implementation of lung cancer screening. Statistical analyses were carried out using R version 4.0.4 or later software (R Foundation for Statistical Computing). Categorical variables were compared using the χ^2^ or Fisher exact test, and continuous variables were compared using Welch’s *t* test. A *P* value of < .05 was considered significant.

## Results

A total of 430 patients, 150 from PANOPTIC and 280 from ORACLE, met inclusion criteria. Table 1 provides patient demographic and radiologic characteristics for each trial cohort. The prevalence of malignancy was 14%. The two cohorts were similar overall; however, the ORACLE cohort was slightly older, included a higher proportion of female patients, and demonstrated a smaller average nodule diameter.

**Table 1:** Patient Demographic Characteristics by Study

Characteristic	Overall (N = 430)	PANOPTIC (n = 150)	ORACLE (n = 280)
Diagnosis			
Benign	368 (86)	126 (84)	242(86)
Cancer	62 (14)	24 (16)	38 (14)
Age, mean (SD), y	66.6 (9.4)	64.5 (10.3)	67.8 (8.7)
Sex			
Female	242 (56)	69 (46)	173 (62)
Male	188 (44)	81 (54)	107 (38)
Diameter, mm^a^	12.4 (4.2)	13.2 (4.7)	12.0 (3.9)
Nodule location			
Other	235 (55)	81 (54)	154 (55)
Upper	195 (45)	69 (46)	126 (45)
Spiculation	58 (13)	26 (17)	32 (11)
Tobacco use status			
Current	103 (24)	32 (21)	71 (25)
Former	216 (50)	77 (51)	139 (50)
Never	111 (26)	41 (27)	70 (25)
Previous history of cancer			
Yes	23 (5.3)	10 (6.7)	13 (4.6)
No	406 (94)	139 (93)	267 (95)
Unknown	1 (0.2)	1 (0.7)	0 (0)
Solitary vs multiple nodules			
Multiple	229 (53)	84 (56)	145 (52)
Single	201 (47)	66 (44)	135 (48)
Emphysema	131 (30)	35 (23)	96 (34)
Screen detected	69 (25)	NA	69 (25)
Mayo SPN risk, mean (SD)	0.2 (0.1)	0.2 (0.1)	0.2 (0.1)

Data are presented as No. (%) unless indicated otherwise. ORACLE = Observational Registry Study to Evaluate the Performance of BDX-XL2 in Routine Clinical Use; PANOPTIC = Pulmonary Nodule Plasma Proteomic Classifier; SPN = solitary pulmonary nodule.

**Figure 1:**
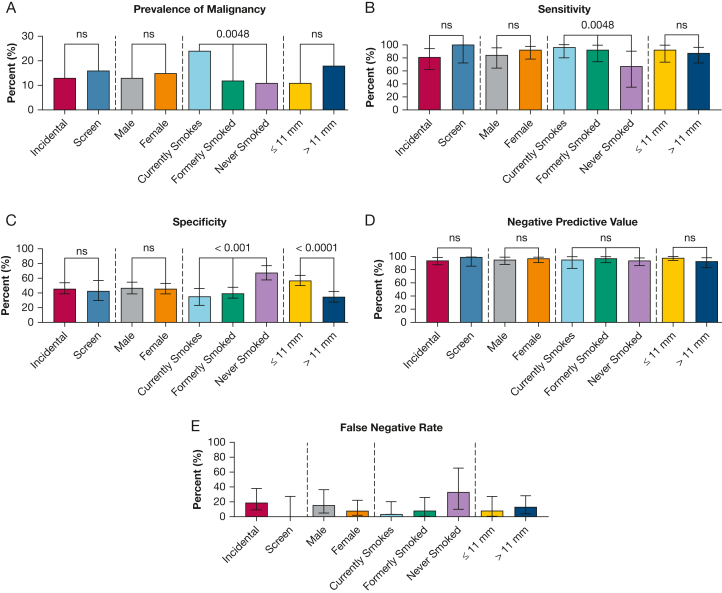
A-E, Bar graphs showing biomarker performance across subgroups: prevalence of malignancy (A), sensitivity (B), specificity (C), negative predictive value (D), and false-negative rate (E). ns = nonsignificant.

Two hundred eleven incidental vs 69 screen-detected nodules were included. No difference in performance of the IC between groups was found (NPV, 94% vs 100%; *P* = .58) (Fig 1). The combined cohort was 54% female and the performance did not differ by sex (NPV, 97% male vs 95% female; *P* = .7023). One hundred three patients currently used tobacco, 216 patients formerly used tobacco, and 111 patients had never used tobacco. Evaluation of the IC demonstrated higher sensitivity and lower specificity in those who had ever used tobacco compared with those who had never used tobacco (94% vs 67% [*P* = .0218] and 39% vs 68% [*P* < .001], respectively); however, the NPV was not different between groups (97% vs 94%; *P* = .43). The median nodule diameter was 11 mm. Evaluation of the IC based on size of ≤ vs > 11 mm demonstrated similar sensitivity (92% vs 87%; *P* = .69); however, specificity was higher for nodules of ≤ 11 mm (57% vs 35%; *P* < .001). Nevertheless, the NPV was similar between the two groups (98% vs 93%; *P* = .105).

## Discussion

The initial validation study for the IC showed strong performance of the biomarker to distinguish benign from malignant incidentally detected nodules.^6^ The addition of the ORACLE cohort allowed us to evaluate the performance of the biomarker in a screen-detected population. The IC showed excellent sensitivity and NPV in this population. Under current practice guidelines, screen-detected nodules of ≥ 8 mm would fall into lung imaging reporting and data system (LungRADS) category 4A or 4B and would require further evaluation with short-interval CT scan, PET/CT imaging, or biopsy.^7^ Incorporation of the IC into a screening population could decrease the rate of unnecessary PET/CT imaging and biopsies by reclassifying likely benign nodules.

Women with a diagnosis of lung cancer are more likely to have never smoked, and management of pulmonary nodules may differ between male and female patients.^8^^,^^9^ This analysis demonstrated equally strong performance of the IC in male and female patients, which would allow both sexes to be routed to a surveillance strategy more accurately without delaying diagnosis of lung cancer.

Tobacco use and nodule size are both well-established risk factors for malignancy and are incorporated into clinical risk prediction models.^3^ The IC showed strong sensitivity and NPV in patients who currently and formerly used tobacco. Although, the sensitivity of the IC decreased for those who never used tobacco, the specificity markedly increased. The specificity of the IC decreased significantly for larger nodules. Because size is responsible for 30% of the IC result,^10^ it is understandable that the test produced more indeterminate results for larger nodules. However, because the IC still showed a high NPV for larger nodules, negative results are still useful for identifying benign disease.

This study has several limitations. First, several of the subgroups included relatively small numbers for analysis. Second, benign status was determined for both studies based on 12 months of CT scan results stability, rather than the standard 24 months, although extended follow-up of the PANOPTIC cohort demonstrated that all nodules classified as benign at 12 months remained benign at 2 years.^10^

In conclusion, this subgroup analysis demonstrated a high NPV for the IC that did not differ based on screen detection, sex, smoking status, or nodule size. These results suggest that the IC may be used effectively in a broader range of patient populations than previously reported.

## Funding/Support

The study was supported by Biodesix, Inc.

## Financial/Nonfinancial Disclosures

The authors have reported to *CHEST* the following: J. S. K. discloses consulting/advisory (Ambu, Biodesix, Inc., Boston Scientific, Cook, Intuitive, Level Ex, Medtronic, Pinnacle Biologics, Pulmonx), research funding (Lung Therapeutics, PrognomiQ), honoraria (Pinnacle Biologics), speaker’s bureau (Biodesix, Inc., Veracyte), stock ownership (Doximity), and travel/accommodations/expenses (Auris); M. A. P. discloses honoraria (Medtronic, Philips, Astra Zeneca), consulting (Medtronic, Philips, Intuitive, Johnson & Johnson, Pfizer, Noah Medical, Galvanize Therapeutics, PreView Medical), research funding (Intuitive, Medtronic, Philips, Biodesix, Inc.), speaker’s bureau (Johnson & Johnson, United Therapeutics, Biodesix, Inc.), and travel/accommodations/expenses (Intuitive, Johnson & Johnson, Astra Zeneca, Pfizer, Noah Medical); T. P. discloses employment (Biodesix, Inc.); G. A. S. discloses research funding (Biodesix, Inc.) and consulting (Biodesix, Inc.). None declared (K. J. L.).
